# Influence of gold nanoparticles on collagen fibril morphology quantified using transmission electron microscopy and image analysis

**DOI:** 10.1186/1471-2342-6-4

**Published:** 2006-05-31

**Authors:** Mark A Haidekker, Lisa W Boettcher, Jonathan D Suter, Rebecca Rone, Sheila A Grant

**Affiliations:** 1 University of Missouri-Columbia, Department of Biological Engineering, Columbia, MO 65211, USA

## Abstract

**Background:**

Development of implantable biosensors for disease detection is challenging because of poor biocompatibility of synthetic materials. A possible solution involves engineering interface materials that promote selfassembly and adhesion of autologous cells on sensor surfaces. Crosslinked type-I collagen is an acceptable material for developing engineered basement membranes. In this study, we used functionalized gold nanoparticles as the crosslinking agent. Functionalized nanoparticles provide sites for crosslinking collagen as well as sites to deliver signaling compounds that direct selfassembly and reduce inflammation. The goal of this study was to obtain a quantitative parameter to objectively determine the presence of crosslinks.

**Methods:**

We analyzed TEM images of collagen fibrils by two methods: Run length analysis and topology analysis after medial axis transform.

**Results:**

Run length analysis showed a significant reduction of the interfibril spaces in the presence of nanoparticles (change of 40%, P < 0.05), whereas the fibril thickness remained unchanged. In the topological network, the number of elements, number of branches and number of sides increased significantly in the presence of nanoparticles (P < 0.05). Other parameters, especially the number of loops showed only a minimal and nonsignificant change. We chose a ratiometric parameter of the number of branches normalized by the number of loops to achieve independence from gross fibril density. This parameter is lower by a factor of 2.8 in the presence of nanoparticles (P < 0.05).

**Conclusion:**

The numerical parameters presented herein allow not only to quantify fibril mesh complexity and crosslinking, but also to help quantitatively compare cell growth and adhesion on collagen matrices of different degree of crosslinking in further studies.

## Background

Implantable biosensors suffer from biofouling and fibrous encapsulation. The key to success with long term implantable sensors lies in integrating the sensor with the surrounding tissue to avoid the immune response. For example, intravascular devices could be coated with autologous endothelial cells to hide the material from the immune system [1,2]. Since these cells would not easily adhere to the biosensor material, a collagen layer would facilitate cell adhesion [3]. Furthermore, covalent crosslinking of the collagen increases stability of the collagen layer [4] and improves cell adhesion and proliferation [5], likely aided by the nanoscale substrate texture [6]. Recent work has focused on functionalized gold nanoparticles as crosslinking agents for collagen and as a facilitator for cell migration and ingrowth [7]. In addition, some studies indicate that increased substrate stiffness influences cell adhesion [8,9]. It is conceivable that enhanced crosslinking may stiffen the network and enhance cell ingrowth through the link between crosslinking and biomechanical properties.

The purpose of this study was to develop an objective image analysis method to quantify the degree of crosslinking of type-I collagen fibrils in the presence or absence of gold nanoparticles. On visual inspection of transmission electron images (TEM), differences could be seen where fibrils in the presence of nanoparticles subjectively appeared disordered and shaped in a more complex manner. We wanted to find a numerical parameter that describes the differences in morphology, therefore allowing to differentiate fibrils in the presence of nanoparticles from control fibrils in the absence of nanoparticles, and eventually promoting further studies where cell growth and adhesion may be related to this parameter.

## Methods

### Collagen fibril formation

Gold nanoparticles of 10 nm average diameter, functionalized with a coating of gum arabic, were generously provided by Dr. Katish Katti, University of Missouri-Columbia. Gum arabic coating provides reaction sites for crosslinking with collagen [10]. Control collagen matrix was formed by mixing 160 μl PureCol™ (3.2 mg/ml bovine collagen solution, Inamed) with 650 μl water and 184 μl PBS. Collagen matrices in the presence of gold nanoparticles were created from 160 μl PureCol, 600 μl water, 240 μl PBS and 250 μl of aqueous gold nanoparticle suspension. Actual fibril formation was initiated by gradually raising both the temperature and pH of the collagen solution simultaneously. The temperature of the solution was increased to 37°C in a water bath. The pH was increased from 2.5 to 7.4 by adding phosphate buffer solution (PBS). This process of fibril formation was carried out over 4 hours. Final collagen was at a concentration of 0.5 mg/ml. The suspension of functionalized gold nanoparticles was added in a 1+4 volumetric ratio (one part gold, 4 parts collagen fibril solution).

### Transmission Electron Microscopy (TEM) sample preparation

Samples of both the collagen control and collagen-nanoparticle solution were prepared by placing 10 μm of the sample on a TEM carbon grid, which was then allowed to air dry for 5 minutes. The sample grid was lightly rinsed with distilled water and dried with filter paper. A drop of 5% uracyl acetate solution was then placed on the grid, and the grid was slightly dried along the edge using filter paper after 10 minutes. This method ensured that the water content of the collagen fibers remained as constant as possible. Images of the matrix were acquired with a JEOL 1200EX transmission electron microscope at 80 kV. Images were printed on Film and scanned on an Epson Expression 800 scanner at 3600dpi and 16 bit/pixel to obtain digital images for analysis.

### Image preprocessing and segmentation

The resulting images were scaled to a size of approximately 1000 by 1000 pixels while retaining the aspect ratio. Binary images were created through a filtering and segmentation process as follows: The scanned image was inverted to obtain bright features on a darker background. Four iterations of a grayscale rank filter, where each pixels is replaced by the median of its 3 by 3 neighborhood, were applied to reduce noise. Background inhomogeneities were then removed by creating a severely blurred image (convolution with a circular kernel of 120 pixels diameter) and by subtracting this blurred image from the rank-filtered image. The image was then binarized by applying Otsu's method [11]. The resulting binary image now contained the fibrils as well as the nanoparticles as white features over a black background. The nanoparticles were then eliminated with a feature size filter, which removed any connected region of less than 150 pixels from the image. The remaining features were finally filtered through morphological closing, so that any small interior holes or fissures disappeared.

### Image analysis

Two different methods were devised to quantify fibril shape complexity. The first method is based on the analysis of run lengths. In this method, the image is scanned line by line in a similar manner as a fax is being scanned for transmission. In a fax, it is much more efficient to transmit the information "100 black dots" than actually transmitting 100 black dots. This information is called a run. Probability histograms of black and white run lengths were created. Our hypothesis was that curved and self-intersecting fibrils (higher feature complexity due to higher degree of crosslinking in the presence of nanoparticles) lead to a higher probability of shorter black runs, while not influencing the white runs (corresponding to unchanged fibril width).

The second method was based on the medial axis transform (MAT) [12]. The MAT erodes features to their central ridges, i.e. their skeletons. The resulting skeletonized image can be analyzed topologically, i.e. in terms of the number of nodes, endpoints, links (connections between nodes), branches (connection between a node and an endpoint), and loops. Our hypothesis is that a more complex shape, caused by curled and self-intersecting fibrils, results in a higher number of nodes and a higher number of loops.

### Statistical analysis

Statistical analysis was performed by using Graphpad Prism 4.01 (Graphpad, San Diego). The images were divided into two equally-sized groups (with and without nanoparticles, n = 10 each). The parameters obtained for each group were tested for normal distribution (Kolmogorov-Smirnov-test) and, after passing the normality test, subjected to the t-test to test the hypothesis that the group means are different.

## Results

**Figure 1:**
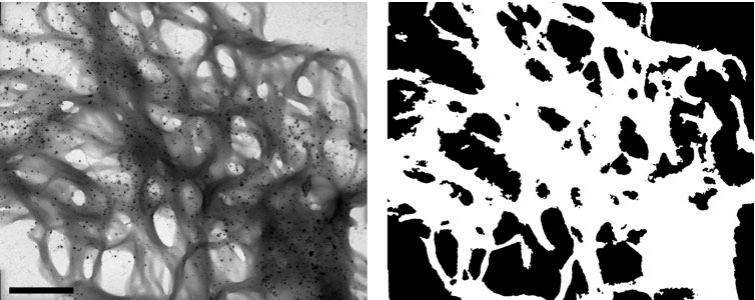
Segmentation of the fibrils. After background removal and segmentation, small particles (nanoparticles showing as black dots in the left image) have been removed. The scale bar corresponds to approximately 1 μm.

**Figure 2:**
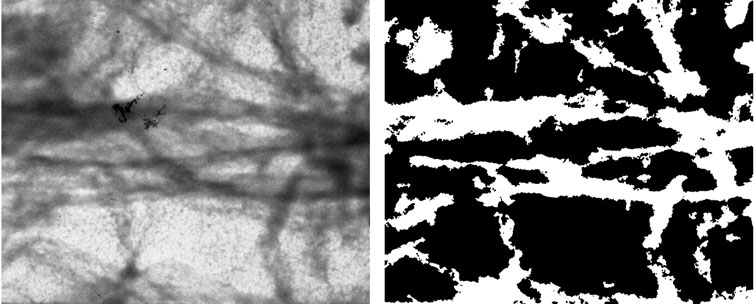
Result of the segmentation process in a representative control image.

Typical images with fibrils in the presence and absence of nanoparticles with their corresponding segmentation results are shown in Figures 1 and 2, respectively.

**Figure 3:**
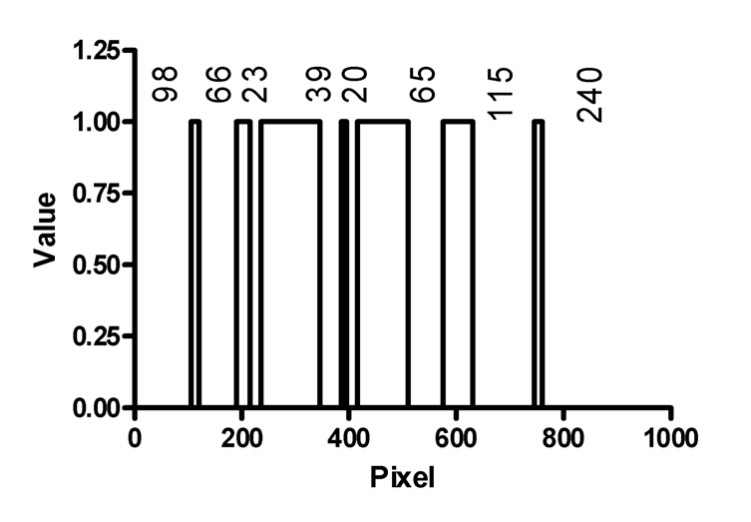
Runs along a horizontal scanline at the top of the segmented image in Figure 1 (right). The numbers above the black runs indicate the length of the individual runs.

**Figure 4:**
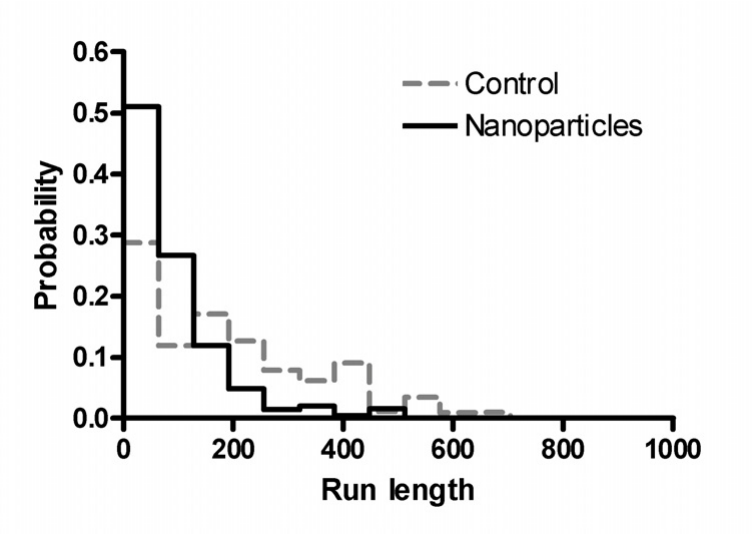
Run length histograms of the black (background) runs of a nanoparticle image an a control image. The higher complexity of the structure in the presence of nanoparticles leads to shorter runs. Consequently, the control histogram is broader and shifted towards longer runs.

Figure 3 shows the runs along one horizontal scanline close to the top of the segmented image in Figure 1. Black runs (value of zero) in Figure 3 correspond to background runs, while white runs (value of 1) correspond to fibril runs. Runs were grouped into 16 equally-sized bins (1 pixel to the maximum run in the respective image), and collected over all scanlines. Two resulting typical run length histograms for a nanoparticle image and a control are displayed in Figure 4.

**Figure 5:**
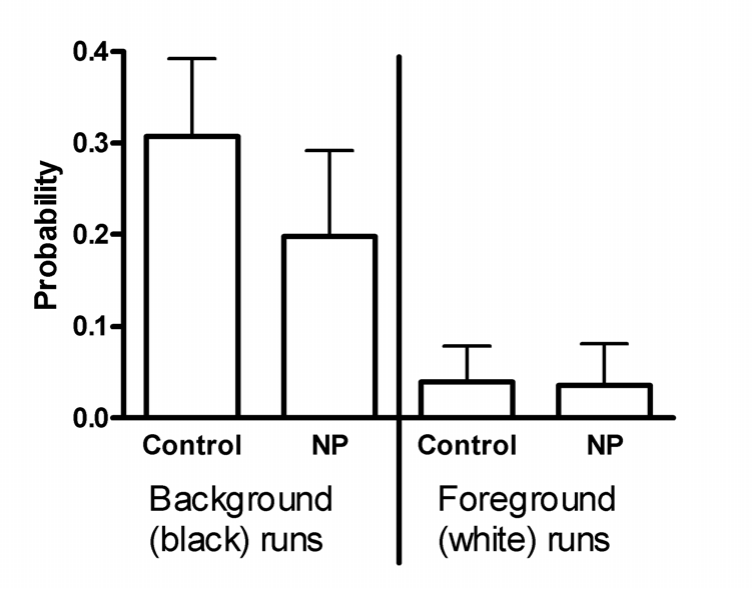
Statistical comparison of the probability that any run belongs to the 75% longest runs. Error bars indicate standard deviation. In the control images, background runs are 40% more likely to belong to the group of longer runs (P < 0.05) than in the nanoparticle images. Foreground runs do not show this difference.

One possible metric for the quantitative description of the fibril complexity is the overall probability per image that an arbitrary run lies in the upper 75% of possible run lengths (i.e. falls into bins 4 to 16). The comparison of this metric over all 20 images (10 images with nanoparticles, 10 controls) showed that a background run was 40% more likely to be in the longer 75% of the runs in the control image than in the nanoparticle image. This difference was statistically significant (t-test, P < 0.05). A foreground (fibril) run was 11% more likely to be in the longer 75% of the runs in the control image than in the nanoparticle image, but this difference was not statistically significant (Figure 5).

**Figure 6:**
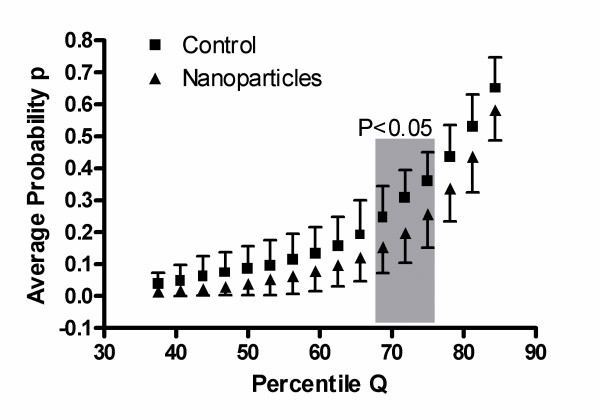
Average probability p (error bars indicate standard deviation) that a run falls into the Q longest runs. The gray shaded area indicates the percentile region where the difference between control images and nanoparticle images is statistically different.

Consistent with our hypothesis, the more complex shape of the fibrils in the presence of nanoparticles leads to shorter background runs. The difference can be quantified by computing the probability of the occurrence of long runs. The selected percentile of 75% was not chosen arbitrarily, but it was the percentile of highest specificity: The relative difference at very low percentiles was low, because the overall probability of very long runs was low. The difference increased with increasing percentile, but random influences increased as well (Figure 6).

### Skeleton analysis

**Figure 7:**
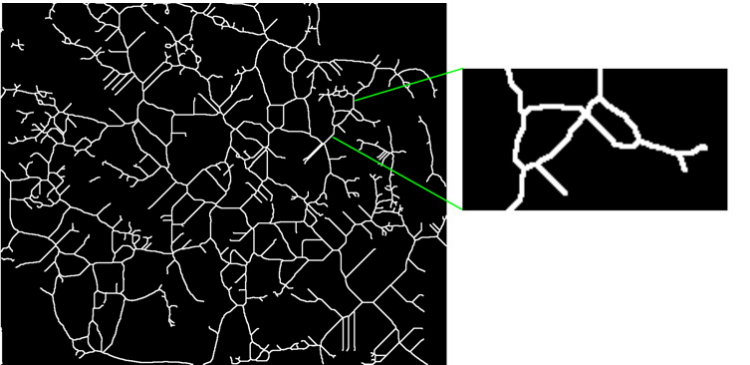
Skeleton of the segmented image in Figure 1. The magnified section (right) shows two loops, 11 links (three of them connected to nodes outside of the field of view), three branches, seven nodes, and three endpoints.

The medial axis transform of the segmented image in Figure 1 is displayed in Figure 7. Out of several topological parameters, only the number of elements, the number of branches, and the number of sides (branches and links) were significantly different between control images and nanoparticle images (Table 1). In addition, the average length of the branches was significantly shorter in the nanoparticle images (34 ± 34 pixels) than in the control images (91 ± 76 pixels, P < 0.05). However, this metric is not scaling-independent.

The number of loops showed the smallest difference between nanoparticle and control images. A ratiometric number was devised by dividing the number of loops by the number of branches. This value was approximately 2.8 times higher in the control group (P < 0.05).

## Discussion

In recent years, efforts focused on creating functionalized surfaces for implants (see e.g. [13] for review). Collagen plays a key role in this process as a substrate to grow cells on. The gold nanoparticles used in this experiment were synthesized and stabilized with a thin film of gum arabic. Gum arabic is a mixture of branched polysaccharides andf glycoproteins that contains many functional sites for crosslinking or drug delivery [10]. Polysaccharides have been shown to be a useful class of biomaterials and typically have good biocompatibility [10]. Collagen fibrils contain amine and carboxyl sites, and are capable of nonspecific hydrogen bonding [14]. Due to the complex nature and variable structure of the gum arabic, the exact binding mechanism is still under investigation. However, we speculate that the nanoparticles bind either by nonspecific hydrogen absorption to the fibrils or by NH_2_/COOH cross-links. Objective, quantitative image analysis methods to determine the degree of crosslinking may be a helpful tool in this process.

Two different unsupervised methods to quantify fibril shape complexity and therefore fibril crosslinking in TEM images were devised in this study. The first was based on the analysis of black (background) and white (fibril) runs in the segmented image. It was hypothesized that the white runs do not show any statistical difference between nanoparticle and control images as the fibril width remains unchanged. The black runs, however, would be shorter in average because curling and self-intersection reduces the average size of the gaps between the fibrils. This hypothesis was supported by our data. A single metric to describe the overall fibril shape in one image was presented, which showed statistically significant differences between nanoparticle and control images. The second analysis method was based on the medial axis transform which provides topological parameters. Several topological parameters were found where a statistically significant difference between the two groups existed. Consistent with our initial hypothesis, those parameters which showed a significant difference (number of elements, number of branches, number of sides) were higher in the nanoparticle images compared to the control images. This indicates a higher complexity of the skeletonized shape.

The medial axis transform is a popular method to quantify network-like structures. For example, confocal images of fluorescently stained DNA and fibrin networks were analyzed for differences of topological and size parameters associated with cystic fibrosis [15]. Shah et al introduce a novel segmentation method for rod-like structures, which was specially developed for very thin networks at the high contrast typical for fluorescence. At higher magnifications, as provided by electron microscopy, the contrast is lower, and the structures become thicker. Morphological analysis has been found to provide significantly different values for segment length, number of segments, and segment orientation when cancer cells were treated with transforming growth factor α [16], a study based on scanning electron microscopy images of the cell's keratin filament network. The most crucial step for the analysis of this type of images is the segmentation step, where the features (fibers) are separated from background. The algorithm presented by Shah *et al *[15] inherently performs the segmentation, whereas no segmentation details are given by Beil et al [16]. Performance comparison with the methods that we presented in this study is therefore difficult. On the basis of our data, however, we conclude that our relatively straightforward approach to fibril segmentation yields sufficiently reliable results.

Shape analysis needs to be robust against various transformations. A curved shape remains – for the human eye – the same shape even if it is rotated and scaled. It is important that any metric reflects this tolerance of human perception against affine transformations. In addition, changes in magnification have a strong impact on length metrics. Therefore, studies using size-based parameters (such as e.g. [15] and [16]) must be particularly carefully designed to ensure unchanged imaging parameters throughout the study.

In this study, it was also important to ensure that the metric was not be affected by the absolute number of fibrils in the image. This is particularly important as fibrils in the presence of nanoparticles tend to cluster. For example, determining the relative segmented fibril area normalized by the total image area yields significantly higher values in the nanoparticle group (P < 0.0001). However, the segmented relative fibril area is not a shape measurement, and therefore not acceptable. Run-length histograms are affected by the total number of fibrils as well. The presence of a higher number of fibrils leads to shorter runs in average. For this reason, we devised a relative parameter – the probability of a run to be in the larger 75% of all runs. This parameter now becomes independent of affine transformations and even hypothetical tiling of the same image (simulation of more fibrils). The same consideration gave us the idea of using a ratiometric parameter in the analysis of fibril topology. While topological parameters are by nature invariant over affine transformations, the addition of more fibrils would change the parameters. Since the average number of loops is almost the same between nanoparticle and control images (less than 14% difference, and with a reverse trend), normalization of a strongly varying parameter (in this case, the number of sides) by the number of loops would yield a parameter that is independent from the overall fibril density. The number of sides divided by the number of loops is therefore our preferred metric to describe overall fibril shape.

The use of shape analysis instead of the analysis of size or density is particularly important, since water content of the collagen fibers may change because of evaporation in spite of the fibril treatment. Water content affects fibril length and density distribution. However, since the amount of drying is the same for control and nanoparticle networks, our comparative measurements will not be affected. In addition, we were careful to use size-independent metrics instead of fibril length, for example. Consequently, our proposed metrics would be robust against some change of water content.

The robustness of the parameters that we analyzed in this study is further supported by the wide variation of the fibril density in the images even within one group. The electron microscope images were taken by eye without consideration of future image analysis. As a consequence of this large intra-group variation, values between the groups overlap. We believe that a complete separation of the values between nanoparticle and control groups would be possible provided that the microscope field of view was selected to provide approximately matched fibril density. However, considering the huge variation of the fibril image, the parameters that we presented in this study are very robust and therefore well suited in the analysis of collagen fibril shape.

The presented image analysis methods rely on standard image operations. It is therefore possible to easily reproduce the image processing steps with most image analysis programs, even freely available ones such as the popular ImageJ . On the long run, the parameters presented in this study may serve as a tool to further quantify the ability of crosslinked collagen to increase cell ingrowth and proliferation. For example, numerical parameters such as cell density or cell morphology could be correlated with the fibril crosslinking parameter. This would allow even more accurate design of collagen matrices to support stable biocompatible cell layers.

## Conclusion

We presented two numerical parameters to quantitatively describe the complexity of collagen fibril morphology. The complexity parameters showed significant differences between TEM images of collagen fibrils crossinked with functionalized gold nanoparticles and control images without gold nanoparticles. Since it has been hypothesized that a complex collagen type-I morphology may provide some of the functionality of collagen type IV, this parameter may be used to quantitatively relate cell growth and proliferation on the collagen substrate to the collagen morphology, thus enabling to optimize the collagen substrate in further studies.

## Competing interests

The authors declare that they have no competing interests.

## Authors' contributions

M.H. refined the image analysis process, performed the actual image analysis and wrote the major part of the manuscript. L.W.B. was responsible for providing fibril samples and performing electron microscopy. In the course of a graduate class project in a biomedical imaging class, R.R. developed the basic algorithm for run length quantification, and J.D.S. developed the basic algorithm for morphological quantification. S.G. provided the methods to create collagen fibers and use functionalized nanoparticles to enhance crosslinking.

## Pre-publication history

The pre-publication history for this paper can be accessed here:



## Figures and Tables

**Table 1:** Selected topological parameters (all values are given as means ± standard deviation).

Topological parameter	Control images	Nanoparticle images	P
Number of Elements	7.5 ± 5.89	13.8 ± 6.34	0.033
Number of Branches	94.9 ± 53.7	155.8 ± 45.8	0.014
Number of Sides	239.2 ± 111.0	355.7 ± 125.1	0.041
Number of holes	16.3 ± 10.6	14 ± 17	n.s. (0.7)
Holes relative to branches	0.22 ± 0.14	0.08 ± 0.09	0.022
